# Why is a small sample size not enough?

**DOI:** 10.1093/oncolo/oyae162

**Published:** 2024-06-27

**Authors:** Ying Cao, Ronald C Chen, Aaron J Katz

**Affiliations:** Department of Radiation Oncology, University of Kansas Medical Center, Kansas City, KS, United States; Department of Radiation Oncology, University of Kansas Medical Center, Kansas City, KS, United States; Department of Radiation Oncology, University of Kansas Medical Center, Kansas City, KS, United States; Department of Population Health, University of Kansas Medical Center, Kansas City, KS, United States

**Keywords:** sample size, statistics, data analysis, random sample, simulation

## Abstract

**Background:**

Clinical studies are often limited by resources available, which results in constraints on sample size. We use simulated data to illustrate study implications when the sample size is too small.

**Methods and Results:**

Using 2 theoretical populations each with *N* = 1000, we randomly sample 10 from each population and conduct a statistical comparison, to help make a conclusion about whether the 2 populations are different. This exercise is repeated for a total of 4 studies: 2 concluded that the 2 populations are statistically significantly different, while 2 showed no statistically significant difference.

**Conclusions:**

Our simulated examples demonstrate that sample sizes play important roles in clinical research. The results and conclusions, in terms of estimates of means, medians, Pearson correlations, chi-square test, and *P* values, are unreliable with small samples.

## Teaser

Clinical studies are often limited by resource availability that results in constraints on sample size. This study used simulated data to illustrate study implications when the sample size is too small.

## Sample and sample size

A sample comprises the individuals from whom we collect data and represents a share of the population (*N*) for whom we want to draw conclusions (eg, women breast cancer).

The sample size (*n*) is the number of individual people, experimental units, or other elements included in a sample, and is a central concept in statistical applications to clinical research. Given that researchers often have limited resources (financial and personnel) and time to conduct a study, it is not feasible to collect data from an entire population and, in some cases, only possible to obtain information from a seemingly small sample of individuals.

## What is a “small” sample size?

There is no universal agreement, and it remains controversial as to what number designates a small sample size. Some researchers consider a sample of *n* = 30 to be “small” while others use *n* = 20 or *n* = 10 to distinguish a small sample size.

“Small” is also relative in statistical analysis. For example, in genome-wide association studies and microbiome research, although the sample size (*n*) is often in the hundreds or even thousands of observations, the number of markers (*p*) of interest (eg, single-nucleotide polymorphisms) is typically in the hundreds of thousands, creating a “large *p* small *n*” conundrum that necessitates the use of advanced statistical techniques for analysis.^1^

## What happens with a small sample size and why is it not ideal?

**Figure 1.**
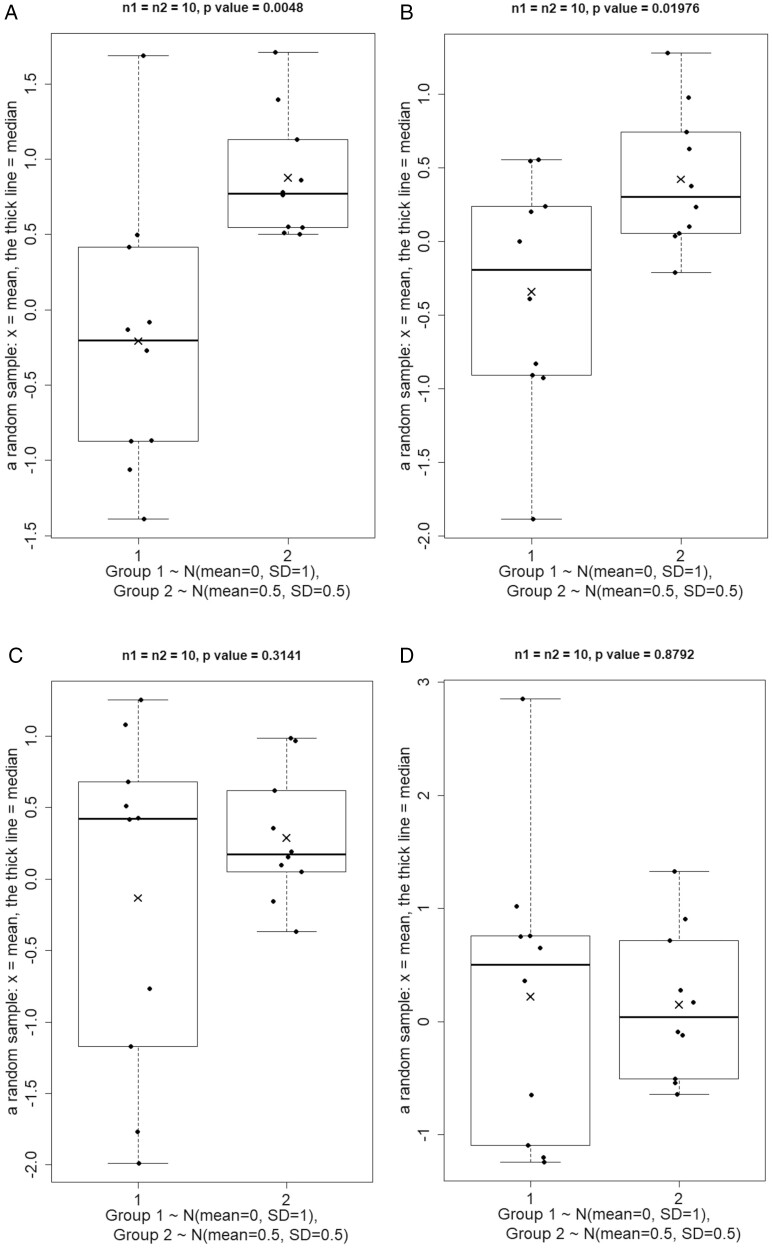
(A) Two random samples of *n* = 10 each were drawn from 2 normally distributed populations each with *N* = 1000. Population 1 has means 0 and SD 1, and population 2 has mean 0.5 and SD 0.5. (B-D) Images illustrate new random samples using the same methodology as in panel (A).

To illustrate some points, we use simulated data representing 2 different theoretical populations (group 1 and group 2)^2^ with a normal distribution for each of the populations (*N* = 1000 for each). Group 1 population has an asymptotic mean = 0 and SD = 1, while the group 2 population has an asymptotic mean = 0.5 and SD = 0.5. This is the entire population and therefore represents the “truth.” Now we randomly select 10 values (10 data points) from each of the normal distributions and perform a (nonparametric) Wilcoxon rank-sum test (also known as Mann-Whitney *U* test) to examine whether both groups come from the same population or have the same shape. We repeat this exercise multiple times (Figure 1).

These 4 results do not support a firm conclusion as to whether the 2 population distributions are either statistically the same or different. Why? Because in 2 of the random samples drawn, as shown in Figure 1a, 1b, the median values differ between the 2 groups, suggesting the population distributions are significantly (*P* value < 0.05) different; still, in the other 2 random samples, as shown in Figure 1c, 1d, the medians are close together, which suggests the population distributions are similar (the *P* values are much larger than .05).

Results from further simulations (not shown) demonstrate that once the sample size reaches *n* = 50, the results from the Wilcoxon rank-sum test (with continuity correction) begin to approach those of the 2-sample *t*-test (with Welch correction for unequal variances), which indicates that the randomly drawn samples are starting to follow a normal distribution. As the sample size increases, the results of the Wilcoxon rank-sum test and 2-sample *t*-tests continue to converge. This yields an explicit confirmation of the large sample theory (asymptotic approximation).

These observations are directly relevant to clinicians and clinical research. For example, an investigator wants to compare the survival outcomes of patients with stage 1 lung cancer treated with lobectomy or stereotactic body radiation therapy. With small sample sizes (eg, 10 patients in each treatment group), there can be random variation in the results; thus, multiple studies of small sample sizes might provide different/opposite findings. With larger sample sizes, such random variation would be reduced and thereby provide more valid results.

This same concept also applies to estimates of other statistics, including the Pearson correlation coefficient *r*, chi-square test, and related *P* values.

## Conclusion

Our simulated example demonstrates that sample sizes play important roles in clinical research. The results and conclusions, in terms of estimates of means, medians, Pearson correlations, chi-square test, and *P* values, are unreliable with small samples. Even when “statistically significant”, small sample size studies might provide spurious results. Thus, caution is needed when interpreting results from small studies.

## Data Availability

The data were created by computer algorithms in software R Core Team,^3^ therefore not directly related to any clinical resources or patients.
